# Editorial (Preface) “Cells/Cells of the Cardiovascular System—Editorial Highlights 2020–2021: The Book Selection”

**DOI:** 10.3390/cells11233898

**Published:** 2022-12-02

**Authors:** Kay-Dietrich Wagner

**Affiliations:** CNRS, INSERM, iBV, Université Côte d’Azur, 06107 Nice, France; kwagner@unice.fr

This introduction provides a preface to the section on “Cells of the Cardiovascular System” in the book entitled “Editor’s Choice Articles in 2020–2021”. Although it remains in its infancy, we have recently experienced a rapid growth of interest in both this section of the journal and the journal itself. Only four papers were published in the section in 2019, while the number increased to 108 in 2020–2021. For the book collecting the Editor’s choice articles, 10 contributions, which are briefly introduced below, were selected. This corresponds to less than 10% of all the articles published in the section during this period. As mentioned in the previous introduction to the Editor’s choice articles under the section “Cells of the Cardiovascular system” [1], the selection was based on personal choice, and all the other published papers that were not selected represent significant contributions to the advancement of scientific research. The following paragraphs highlight my view of the remarkable and most interesting findings and concepts presented in the selected manuscripts.

Peroxisome proliferator-activated receptors (PPARs) are well known as regulators of the metabolism. They are implicated in metabolic and cardiovascular diseases, as well as cancer [2,3,4,5,6,7]. In addition to the defined synthetic agonists and antagonists, which are available to all PPARs, these transcriptional regulators are activated by endogenous fatty acid mediators produced by cyclooxygenase (COX), lipoxygenase, and CYP450 enzymatic pathways [8]. However, our knowledge of endogenous ligands, their tissue sources, and their specificity for the different PPAR forms remains limited. Furthermore, most have been were generated using cell culture or rodent models. Edin and colleagues performed the lipidomic profiling of porcine tissues to identify the sources of endogenous PPAR ligands [9]. This approach was astutely selected, as pigs and humans share many similarities in cardiovascular physiology. The authors measured oxylipins in the cardiac perivascular tissue, aorta, and coronary and pulmonary arteries and identified the coronary artery as the main source of CYP450-derived epoxy fatty acids. Notably, these coronary-artery-derived oxylipins have anti-inflammatory and vasodilatory properties, making them interesting candidates for the treatment of cardiovascular disease. The means by which this knowledge could be translated into potential novel therapeutic approaches remains an open subject for future studies.

In the same Special Issue of *Cells*, entitled “The Role of PPARs in Disease”, Fougerat et al. reviewed the role of PPARs in non-alcoholic fatty liver disease (NAFLD) [10]. NAFLD represents the most frequent liver disease, and with the increasing prevalence of obesity, the number of NAFLD cases worldwide is steadily rising [11,12,13]. NAFLD is characterized histomorphologically by lipid accumulation in the hepatocytes and an inflammatory response in the liver. It can, ultimately, lead to liver fibrosis and cirrhosis, with very limited treatment options. Thus, therapeutic strategies besides the well-known and beneficial life-style changes and reduction in obesity are urgently required. The authors review the pathophysiology, mechanisms of progression, and therapeutic strategies for non-alcoholic fatty liver disease in detail and focus on the role of PPARs in the progression and reversal of the disease. Finally, they provide an overview of the PPAR agonists in current clinical use and experimental novel PPAR agonists for the potential treatment of NAFLD. The review is highly informative in terms of various aspects of cell biology, as well as clinical applications.

Wiersma and colleagues investigated whether cell-free circulating mitochondrial DNA might represent a potential biomarker for atrial fibrillation [14]. Atrial fibrillation (AF) is the most common cardiac tachyarrhythmia and can potentially lead to severe complications, such as stroke, heart failure, and increased mortality [15]. Although episodes of AF are readily detectable through electrocardiographic (ECG) measurements, these methods of verification are not informative in AF-free intervals and do not enable the evaluation of the AF stage. As the authors had previously identified mitochondrial dysfunction as the underlying mechanism of AF, in the present study, they analyzed whether circulating mitochondrial DNA could represent a biomarker for AF. They found that the mitochondrial DNA levels in blood samples correlate with the AF stage and are high, especially in male patients with paroxysmal AF. Furthermore, mitochondrial DNA levels are associated with the recurrence of AF in patients undergoing treatment. These significant observations should stimulate larger clinical trials on the subject and the extension of these measurements to individuals with unrelated diseases who do not have AF, as cell-free circulating mitochondrial DNA might also be indicative of non-AF-related cellular damage. Thus, the specificity of this potential novel biomarker for AF remains to be determined.

Starreveld et al. also published a study on AF in *Cells*. They performed a small pilot study investigating dietary L-glutamine supplementation in patients with AF [16]. The preoperative administration of L-glutamine induces the expression of heat shock proteins (HSPs) [17], which protect the heart against AF [18]. Thus, it appears logical to test whether L-glutamine supplementation might offer protection against AF. The HSP27 levels in the blood samples decreased after 3 months and returned to normal values after 6 months, while the HSP70 levels also diminished after 3 months but remained low after 6 months. Given the nature of this small clinical trial, the cardiac heat shock protein levels could not be evaluated. Whether L-Glutamine dietary supplementation has beneficial effects on AF characteristics remains a subject for future clinical trials, which, hopefully, will be initiated on the basis of this novel publication in *Cells*.

Krishnan and colleagues reviewed the interactions between myocytes, fibroblasts, and adipose tissue in the development of atrial fibrosis and fibrillation (AF) [19]. Coupling between cardiomyocytes and myofibroblasts had been documented in previous literature [20], as had the opposite effect, namely, a mechanoelectrical feedback mechanism in which the contraction of the cardiac tissue influences the fibroblast membrane potentials and secondary cardiomyocyte electrical properties [21]. More recent evidence suggests that adipose-tissue-derived factors, i.e., adipokines, might have beneficial as well as detrimental effects on the development of atrial fibrosis and AF. This informative review summarizes both the electrophysiological and humoral interactions between the different cell types. Scientific understanding of the complex interactions between the different cell types is a pre-requisite for the attainment of deeper insights into the pathophysiology of atrial fibrillation and AF. In contrast to our “classical” cardiomyocyte-centered view of AF, adipose tissue and fibroblasts may come to represent important therapeutic targets for this disease.

In the lung, too, the importance of interactions between cell types differing in their physiology and pathophysiology is increasingly being recognized. Crosstalk between parenchymal, vascular, and immune cells has been described in the literature. Hu et al. reviewed the role of perivascular inflammation in pulmonary arterial hypertension (PAH) [22]. As the inflammatory response correlates with vascular remodeling, hemodynamic parameters, and clinical outcomes [23], the targeting of this inflammatory response might offer novel therapeutic opportunities for the treatment of PAH.

In this review, Hu and colleagues summarized the current state of knowledge on the inflammatory mediators, cytokines, and immune cell types involved in the PAH pathophysiology. They described the effects of these immune mediators on vascular remodeling, as well as the consequences of vascular remodeling, which, in turn, affect the immune cells. This comprehensive review facilitates our understanding of the complex cell–cell interactions in the development and pathophysiology of pulmonary artery hypertension.

To add another layer of complexity, the vessels are also comprised of different cell types, and even the endothelial cells have different characteristics dependent on their localization and function. As a prerequisite for the modification of the vascular phenotype and response, knowledge about the differences between distinct vascular beds is required. Hennigs et al. discussed the heterogeneity of vascular endothelial cells in detail [24]. They introduced the general characteristics of the endothelium and the physiological heterogeneity of endothelial cells, offering a timely introduction to the topic for scientists working in the field. Modifications of the endothelium in response to stress, i.e., inflammation, ischemia, and cancer, are also discussed, and finally, the different strategies used to target distinct endothelial cell populations are reviewed. This work provides an excellent overview and guidance for researchers aiming to achieve selective endothelial cell modifications.

In the same line of thought, it is widely believed that the unfavorable outcomes of chronic ischemic heart disease, myocardial infarction (MI), and dilated cardiomyopathy (DCM) are associated with impaired angiogenesis, and thus, the induction of an angiogenic response might be beneficial and result in functional improvements. Arif and colleagues used a mouse model of DCM and tested the above-mentioned hypothesis by crossing the animals with a line exhibiting enhanced cardiac angiogenesis (microRNA-210 transgenic mice) [25]. As expected, the double-transgenic animals showed enhanced angiogenesis and Vegf expression but, surprisingly, displayed the same morphological signs of DCM and comparable diminished cardiac function to the single-transgenic DCM mice. These data agree with our report on an enhanced angiogenesis model after MI without cardiac functional benefits [26]. These intriguing findings challenge the above-mentioned model and, hopefully, will stimulate further research aiming to unveil the more complex repair mechanisms of the heart. Furthermore, they point to the need to investigate cardiac function in pre-clinical models, in addition to the morphological and molecular parameters.

Similarly, in stroke, most cases are due to an ischemic insult (occlusion of the cerebral arteries) affecting the vessels, and consequently, the major clinical treatment strategies aim to achieve early re-vascularization. In cases where this is not possible or of limited success, severe brain damage might result from a stroke. Thus, in this case, too, novel therapeutic strategies are required. In recent years, glia has attracted increasing attention as modulator of stroke outcomes. Hernández and colleagues reviewed the different types of glial cells together with their bivalent phenotypes, signaling pathways, and potential clinical applications [27]. This excellent review provides a useful overview of the role of glial cells as therapeutic targets in brain ischemia–reperfusion injury and offers new directions for research in the field.

Last but not least, we can note that *Cells*, in addition to publishing manuscripts on different cell types and their interactions in terms of physiology and pathophysiology, also provides also a platform for research on the function of subcellular structures and organelles. The endoplasmic reticulum is increasingly being recognized as an important player in cardiovascular diseases. In general, it is assumed that mild-to-moderate endoplasmic reticulum (ER) stress can induce cell adaptation responses, while chronic or severe ER stress can provoke a pro-apoptotic response contributing to the development of the cardiac disease. Recently, Pires Da Silva et al. reported in *Cells* that SIRT1 protects the heart from ER-stress-induced injury. In mechanistic terms, they related this effect to the induction of eEF2K/eEF2-dependent autophagy [28]. The deacetylase Sirtuin 1 (SIRT1) is known to mediate cardioprotective effects. Surprisingly, the authors found that protective autophagy might be induced in response to ER stress. Sirt1 inhibition diminished this effect and exacerbated cardiac dysfunction. Sirt1 mediates the pro-autophagy cardioprotective mechanism via the activation of the eEF2K/eEF2 pathway. This interesting paper contributes to the creation of a more complex picture of the roles of ER stress and autophagy in the development of cardiac disease.
